# Interfacial Dipole poly(2-ethyl-2-oxazoline) Modification Triggers Simultaneous Band Alignment and Passivation for Air-Stable Perovskite Solar Cells

**DOI:** 10.3390/polym14132748

**Published:** 2022-07-05

**Authors:** He Xi, Zhicheng Song, Yonggang Guo, Weijia Zhu, Lisong Ding, Weidong Zhu, Dazheng Chen, Chunfu Zhang

**Affiliations:** 1 School of Advanced Materials and Nanotechnology, Xidian University, Xi’an 710126, China; 18329743263@163.com (W.Z.); d1446124526@126.com (L.D.); 2 State Key Discipline Laboratory of Wide Band Gap Semiconductor, School of Microelectronics, Xidian University, Xi’an 710071, China; songzhicheng@spic.com.cn (Z.S.); wdzhu@xidian.edu.cn (W.Z.); dzchen@xidian.edu.cn (D.C.); 3 Qinghai Huanghe Hydropower Development Co., Ltd., Xi’an Solar Power Branch, Xi’an 710000, China; guoyonggang@spic.com.cn

**Keywords:** perovskite solar cell, dipole polymer, PEOz, hygroscopic, interfacial engineering

## Abstract

To promote the performance of perovskite solar cells (PSCs), its theoretical power conversion efficiency (PCE) and high stability, elaborative defect passivation, and interfacial engineering at the molecular level are required to regulate the optoelectric properties and charge transporting process at the perovskite/hole transport layer (HTL) interfaces. Herein, we introduce for the first time a multifunctional dipole polymer poly(2-ethyl-2-oxazoline) (PEOz) between the perovskite and Spiro-OMeTAD HTL in planar n-i-p PSCs, which advances the PSCs toward both high efficiency and excellent stability by stimulating three beneficial effects. First, the ether–oxygen unshared electron pairs in PEOz chemically react with unsaturated Pb^2+^ on the perovskite surfaces by forming a strong Pb–O bond, which effectively reduces the uncoordinated defects on the perovskite surfaces and enhances the absorption ability of the resulting PSCs. Second, the dipole induced by PEOz at the perovskite/HTL interface can decrease the HOMO and LUMO level of Spiro-OMeTAD and optimize the band alignment between these layers, thereby suppressing the interfacial recombination and accelerating the hole transport/extraction ability in the cell. Third, the hygroscopic PEOz thin film can protect perovskite film from water erosion by absorbing the water molecules before perovskite does. Finally, the PEOz-modified PSC exhibits an optimized PCE of 21.86%, with a high short-circuit current density (Jsc) of 24.88 mA/cm^2^, a fill factor (FF) of 0.79, and an open-circuit voltage (Voc) of 1.11 V. The unencapsulated devices also deliver excellent operation stability over 300 h in an ambient atmosphere with a humidity of 30~40% and more than 10 h under thermal stress.

## 1. Introduction

Since Miyasaka and co-workers first introduced the organometallic lead perovskite materials as light absorbers in dye-sensitized solar cells [1], hybrid lead halide perovskite solar cells (PSCs) have aroused substantial attention as an emerging next-generation thin-film photovoltaic technology due to their feasibility of low-temperature processability, remarkable PCE and inexpensive fabrication [2,3,4]. Over the past decade, the PCE of PSCs has dramatically advanced from 3.8% in 2009 to the currently certified value of 25.5%, which is steadily approaching the record efficiency (26.7%) of leader single-junction crystalline silicon solar cells, demonstrating great potential for the delivery of cost-effective solar energy [1,5,6,7,8]. The ever-increasing efficiency of PSCs predominantly stems from the unique optoelectronic properties of perovskite materials, including excellent hole/electron mobilities, the long diffusion length of carriers, ambipolar charge transport properties, high absorption coefficient in a wide wavelength region and tunable band gap for tandem cells, which may endow further growth for PCE, beyond those for silicon cells, heading for the theoretical efficiency based on Shockley–Queisser limit [9,10,11,12].

Despite these excellent physical properties and application potentials, PSCs have been suffering from interface loss and the inferior stability of most current perovskite film under real working conditions; both are critical bottlenecks delaying the commercialization of state-of-the-art PSCs technology [13,14,15]. In general, the typical planar PSCs adopt a sandwich-type structure with a perovskite photo-active layer intercalated between two thin charge-transfer layers and electrodes for charge extraction. In this scenario, interfaces play an important role in improving the device’s performance and operational stability. Recent studies have revealed that charge carrier dynamics at the interfaces including charge transport, extraction and recombination, interfacial energy level mismatch, interfacial vacancies and defects arising from weak adhesion between the hole and electron transporting layers and the perovskite film have a significant influence on the PSCs performance and stability [16,17,18,19]. Actually, to some extent, the interface issues for PSCs are closely related to the inferior quality of polycrystalline perovskite films. Recent studies have indicated that the interfacial recombination of charge carriers in most reported PSCs, specifically the nonradiative trap-assisted recombination, is mainly due to the intrinsic crystal growth process of perovskite materials, which includes a large number of vacancies and defects [20,21,22,23]. These defects, especially the under-coordinated lead cations, halogen anion vacancies and lead–halogen antisites located at the interfaces with the charge transport layers and/or at grain boundaries (GBs), are principally caused by structural defects, such as the insufficient coordination between metal cations and halogen anions [4,24,25]. They can induce the generation of localized energy state in ruling the energy level alignment that further leads to severe nonradiative carrier recombination and ion migration, which makes PSCs vulnerable to moisture, oxygen and thermal stresses, resulting in the decrease in the device efficiency along with poor stability [26,27].

Therefore, it is highly desired to passivate those defects located at surfaces and GBs [28,29,30,31]. The aforementioned factors act as a guideline for optimizing PSCs in terms of developing efficient interlayer materials and feasible interfacial engineering through surface passivation that contributes to suppressing the structural defects and improving the quality and chemical stability of perovskite films, which can further help to eliminate the interfacial energy barriers, reduce trap density and nonradiative recombination loss at interfaces, and thus enhancing the transport and extraction ability of charge carriers at the interfaces, targeting to top cell efficiency [32,33]. More recently, various successful interface modification strategies have been proposed and were concerned with solving the above obstacles, which produced a large number of emerging interfacial modifier materials including organic halide salt, i.e., phenylethylammonium iodide (PEAI), phenylmethylamine iodide (PMAI), butylammonium iodide (BAI) and 1-naphthylmethylamine iodide (NMAI) [34,35,36], organic molecules with specific functional groups [37,38,39,40,41], two-dimensional materials [42,43,44] and others [45,46,47] capable to adjust the interface dynamics of charge carriers in PSCs and ultimately boost device performance and operational stability. Among these materials, the dipole molecules, such as poly(2-ethyl-2-oxazoline) (PEOz), polyethoxyvinylimine (PEIE) and polyethyleneimine (PEI), have aroused enough attention because of their versatility and ease to operate features. Their dipole nature has demonstrated that they can not only optimize the interfacial band alignment but also enhance the adhesion between layers, facilitating efficient carrier injection and extraction at interfaces, as well as suppressing carrier recombination [19]. He et al. employed PEOz nanodots as an efficient dipole layer between the electron-collecting layer (PCBM) and the Ag cathode in inverted PSCs. They concluded that the dipole effect caused by the non-ionic PEOz layer can eliminate the contact barrier and decrease the work function of the Ag electrode, which remarkably improved the electron extraction at PCBM/Ag interfaces and the final device performance [48,49]. Recently, Taylor and coworkers integrated PEOz into PEDOT:PSS HTL to tune its energy level and the quality of perovskite film. The results exhibited that the hole trap-state density and noncapacitive current could be effectively reduced and the champion PCE of 17.39% was achieved with the device structure of ITO/PEOz-PEDOT:PSS/CH_3_NH_3_PbI_3_/PCBM/Bphen/Ag [50]. Despite these achievements, we found that PEOz has rarely been employed in the perovskite/HTL interface in n-i-p planar PSCs, which is currently the most popular device model for the exploration of PSCs due to its excellent performance.

In this work, we illustrate for the first time that the dipole polymer PEOz can be employed to passivate the defects on perovskite surfaces and regulate the energy level of spiro-OMeTAD HTL, with a resulting optimization of band alignment for the planar n-i-p structured PSCs. It is found that the oxygen atom in PEOz chemically reacts with coordinatively unsaturated Pb2+ on perovskite films, and thus passivates those uncoordinated Pb2+ defects. Further, the dipole nature of PEOz reduces the HOMO of the Spiro-OMeTAD layer from −5.09 eV to −5.25 eV, resulting in a lower energy barrier between the perovskite and Spiro-OMeTAD layer. Based on these desirable features, further measurements reveal that the dipole PEOz modifier triggers enhanced absorption and photoluminescence lifetime, reduced nonradiative charge carrier recombination, improved charge transport ability as well as enlarged photo-voltage and fill factor (FF) in resulting PSCs. Consequently, the optimized PSC based on PEOz demonstrates a remarkable PCE of 21.86% with a high open-circuit voltage (Voc) of 1.11 V, a short-circuit current density (Jsc) of 24.88 mA/cm^2^, and an FF of 0.79. In addition, the hygroscopic property of PEOz ensures that the perovskite film is highly stable during operation and storage in the ambient air atmosphere via screening water molecules. Therefore, the unencapsulated devices maintain 92.29% of initial efficiency after 300 h of storage in ambient air with relative 30~40% humidity (RH) air. While heated at 85 °C for 10 h in a glovebox, the devices exhibit ca. 17% reduction in efficiency. All these results highlight the multi-functional role of the dipole and hygroscopic PEOz thin film in improving the performance of PSCs.

## 2. Experimental Section

### 2.1. Materials

All the materials were used as received without further purification. Patterned ITO glass substrates (10 ohm·sq^−1^, 2 × 2.5 cm^2^) were purchased from the NSG group. Methylammonium iodide (MAI, 99.8% purity), Formamidinium iodide (FAI, 99.8% purity) and tris [2-(1H-pyrazol-1-yl)-4-tert-butylpyridine] cobalt(III) tris [bis(trifluoromethylsulfonyl)imide] (FK209) were bought from Dyesol Ltd.(Queanbeyan, NSW, Australia). N, N-dimethylformamide (DMF, 99.8% purity) was bought from Aladdin (Shanghai, China). Spiro-OMeTAD was supplied by Xi’an Polymer Light Technology Inc(Xi’an, China). Lead iodide (PbI_2_, 99.999% purity), Lead chloride (PbCl_2_, 99.999% purity) and Stannic oxide (SnO_2_) were obtained from Alfa (Haverhill, MA, USA). Other materials, including Poly(2-ethyl-2-oxazoline) (PEOz, MW ~50,000), trifluoroethanol (TFE, 99.0% purity), isopropanol (IPA, 99.5% purity), chlorobenzene (CB, 99.8% purity), acetonitrile (anhydrous, 99.8% purity), bis-(trifluoromethane) sulfonamide lithium salt (LiTFSI, 96% purity) and 4-tBP (96% purity), were supplied by Sigma-Aldrich (Shanghai, China).

### 2.2. Fabrication and Characterization of Perovskite Solar Cells

ITO glass substrates were cleaned with detergent, deionized water, acetone, and absolute ethanol in the ultrasonic bath for 20 min, respectively. Then, the ITO substrates were dried with nitrogen and treated in a UV-ozone oven for 15 min. A SnO_2_ with a concentration of 3.46 M was spin-coated on ITO substrates at 3000 rpm for 30 s and subsequently annealed at 150 °C for 30 min in air. The substrates with SnO_2_ were then transferred into a glove box filled with highly pure N_2_. After that, the spin-coating procedure of the perovskite layer was performed as follows: First, 1.36 M PbI_2_ and 0.24 M PbCl_2_ were dissolved in the DMF solvent and stirred for 2 h at 75 °C; 70 mg MAI and 30 mg FAI were dissolved in 1 mL IPA with an additional 10 μL DMF. Second, the PbX_2_ precursor solution was spin coated on the top of the SnO_2_-covered substrates at 3000 rpm for 45 s, and then the solution of MAI and FAI was spin coated on top of the PbX_2_ substrates at 3000 rpm for 45 s. Finally, the samples were annealed at 100 °C for 10 min on a hotplate. After cooling down to room temperature, the PEOz in trifluoroethanol solutions (1 mg/mL) were spin coated onto the surface of the perovskite layer at 5000 rpm for 45 s and annealed at 80 °C for 10 min. Next, the hole transport material (HTM) was spin coated on ITO/SnO_2_/MA_0.7_FA_0.3_PbI_3−x_Cl_x_/PEOz at 1000 rpm for 5 s and 4000 rpm for 45 s using the prepared HTM solution. The HTM solution contained 90 mg of Spiro-OMeTAD in 1 mL of chlorobenzene with 45 μL of LiTFSI/acetonitrile solution (170 mg/mL), 10 μL of tBP, and 75 μL of Co(III) complex FK209/acetonitrile solution (100 mg/mL). Finally, 100-nm-thick Ag layers were thermally evaporated as the top electrodes. The area of the device was 0.07 cm^2^ defined by a shadow mask.

### 2.3. Device and Material Characterization

All current density-voltage (J-V) curves were recorded using a Keithley 2400 source meter unit under simulated AM 1.5 G illumination at an intensity of 100 mW/cm^2^ with an XES-70S1 solar simulator. The system was calibrated using a NREL certified monocrystal Si photodiode detector before device testing. Scanning electron microscopy (SEM) images were obtained on a JSM-7800F SEM. The X-ray diffraction (XRD) test was conducted on a Bruker D8 Advance XRD. The UV-visible absorption spectra were measured on a Perkin–Elmer Lambda 950 spectrophotometer. The samples were prepared in the same process of device fabrication. The incident photon-to-current conversion efficiency (IPCE) spectra were recorded using a solar cell quantum efficiency measurement system (SCS10-X150, Zolix instrument. Co., Ltd., Beijing, China). Steady photo-luminescence (PL) and time-resolved photo-luminescence (TR-PL) were measured by using the Pico Quant Fluotime 300 with a 510 nm picosecond pulsed laser. Transient photocurrent (TPC) measurement was performed with a system excited by a 532 nm (1000 Hz, 3.2 ns) pulse laser. Transient photovoltage (TPV) measurement was performed with the same system excited by a 405 nm (50 Hz, 20 ms) pulse laser. A digital oscilloscope (Tektronix, D4105) was used to record the photocurrent or photovoltage decay process with a sampling resistor of 50 Ω or 1 MΩ, respectively. All the measurements of the solar cells were performed under an ambient atmosphere at room temperature without encapsulation.

## 3. Results and Discussion

### 3.1. Interactions of PEOz with MA_0.7_FA_0.3_PbI_3−x_Cl_x_

**Figure 1:**
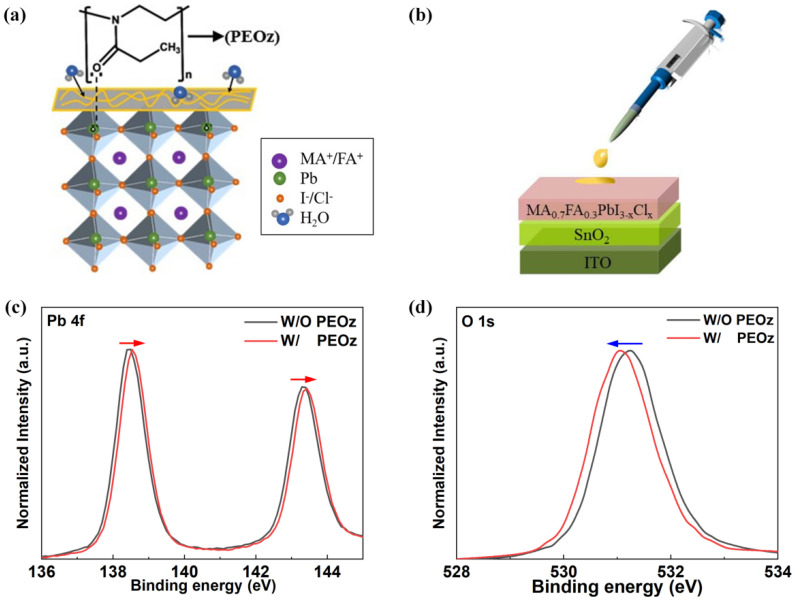
(**a**) Illustration of PEOz passivation mechanism. (**b**) Schematic diagram of PEOz spin coated on perovskite film. (**c**) Core−level Pb 4f and (**d**) O 1s XPS spectra of MA_0.7_FA_0.3_PbI_3__−x_Cl_x_ films with and without PEOz passivation.

As presented in Figure 1a,b, the PEOz in trifluoroethanol solution was deposited on perovskite film by the spin-coating method, and after being annealed at 80 °C for 10 min, the PEOz modified samples were subjected to the follow-up measurements. In order to find the optimal passivation condition, three different concentrations of PEOz solution (0.5, 1, 1.5 mg/mL) were employed in this study. Noting that during the photocurrent density-voltage (J-V) measurements for PSCs based on different concentrations of PEOz with the device structure of ITO/SnO_2_/MA_0.7_FA_0.3_PbI_3−x_Cl_x_/PEOz/Spiro-OMeTAD/Ag, the device with 1 mg/mL PEOz yields the highest PCE of 21.86% because of its outstanding Jsc and FF (Figure S1 and Table S1). So, in this work, we use the optimal PEOz concentration of 1 mg/mL in trifluoroethanol to fabricate the film and device samples for the following characterization and discussion.

To obtain an in-depth understanding of the interaction between the PEOz and perovskite film, X-ray photoelectron spectroscopy (XPS) measurements for pristine perovskite and the perovskite treated by 1 mg/mL PEOz were carried out. For simplicity, the pristine perovskite film and the control cell are named “W/O”, and the ones treated with PEOz are labeled as “W/”. The chemical state changes of the perovskite film induced by PEOz are probed, and the Pb 4f and O 1s spectra are shown in Figure 1c,d, respectively. For pristine perovskite film, two clear core-level Pb 4f characteristic peaks centered at 138.38 eV and 143.30 eV are observed, corresponding to Pb 4f_7/2_ and Pb 4f_5/2_, respectively. While the O 1s peak located at 531.24 can also be detected from the pristine perovskite, which primarily originated from the absorbed oxygen on the perovskite surfaces. After PEOz treatment the two Pb 4f peaks shift toward higher binding energy centered at 138.55 eV and 143.41 eV, and the O 1s peak shifts to lower binding energy by 210 meV, manifesting the increase in the positive charge on the Pb element and negative charge on the O element [51]. We ascribe this phenomenon to the strong coordinate bonding between the O from PEOz chains and Pb^2+^ ions from the perovskite surfaces. It is noted that the O in PEOz contains a lone pair of electrons, which makes it easy to form a chemical bond with Pb^2+^ ions. Moreover, the high electronegativity of O leads to a partial charge transfer from Pb to the PEOz; the proposed working mechanism is schematically presented in Figure 1a [52]. The formation of Pb–O bonds certainly will passivate the uncoordinated surface Pb^2+^ centers on perovskite film and repress the nonradiative recombination of charge carriers [53].

### 3.2. Characterization of MA_0.7_FA_0.3_PbI_3−x_Cl_x_ Films with PEOz

**Figure 2:**
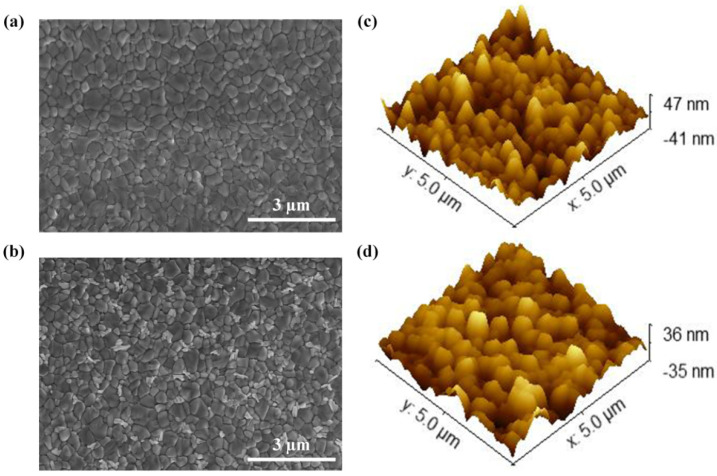
The top view SEM and AFM images of (**a,c**) pristine MA_0.7_FA_0.3_PbI_3−x_Cl_x_ thin film and (**b,d**) MA_0.7_FA_0.3_PbI_3−x_Cl_x_ thin film with PEOz treatment.

**Figure 3:**
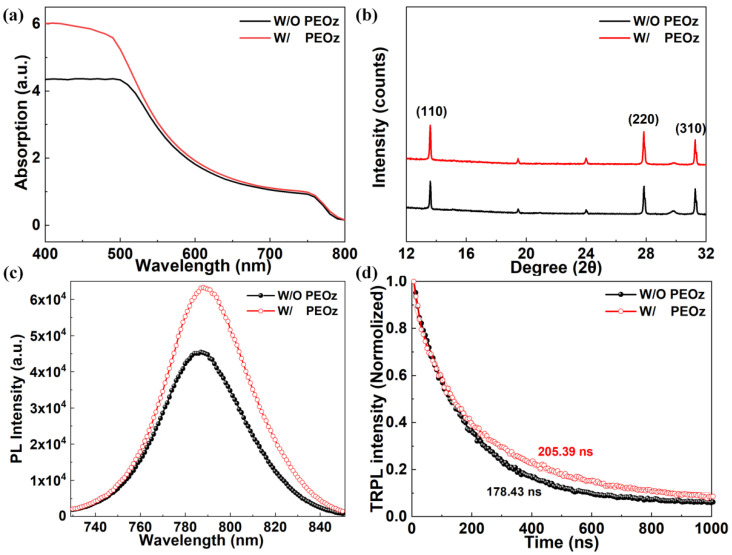
Optical properties and crystal structure of the as−prepared MA_0.7_FA_0.3_PbI_3−x_Cl_x_ films with and without PEOz. (**a**) UV−Vis absorption spectra, (**b**) XRD patterns, (**c**) PL spectra and (**d**) normalized TRPL spectra of the pristine perovskite film and PEOz−treated perovskite film grown on the glass substrates.

The effect of PEOz on the morphological properties of the MA_0.7_FA_0.3_PbI_3−x_Cl_x_ perovskite film is investigated by scanning electron microscopy (SEM) and atomic force microscopy (AFM), respectively. From the top surface SEM images in Figure 2a,b, we can find that both perovskite films present a closely packed surface morphology with triple junction grain boundaries. There is no great difference in grain size or any discernible pinholes observed for the two perovskite films. While after the PEOz treatment, some white extraneous substances with visible contrasts appear at the MA_0.7_FA_0.3_PbI_3−x_Cl_x_ grain boundaries, as indicated in Figure 2b. These substances are most likely assigned to the introduced PEOz since no PbI_2_ diffraction signals are detected in the X-ray diffraction (XRD) patterns for the PEOz-treated perovskite film (see Figure 3b). It should be noted that deep-level defects usually exist in grain boundaries that cause fast non-radiation carrier recombination, so the PEOz located at grain boundaries should contribute to bringing down the boundary defects and related energy losses of the perovskite film and help to realize more compact crystalline grains with smoother surfaces. The AFM results in Figure 2c,d confirm the morphology evolution of the perovskite film with PEOz. The root-mean-square (RMS) roughness values of the MA_0.7_FA_0.3_PbI_3−x_Cl_x_ films decrease from 12.39 nm to 9.99 nm after 1 mg/mL PEOz treatment as a result of the passivation of boundary defects. The smoother perovskite surface with PEOz could enable better interface contacts between perovskite and upper HTLs, providing a more effective channel for carrier separation and extraction.

Next, the influence of PEOz introduction on the optical properties and crystallographic structure of the MA_0.7_FA_0.3_PbI_3−x_Cl_x_ films are studied. Figure 3a shows the UV-vis spectra of the perovskite film with and without PEOz. The PEOz-treated perovskite film obtains higher absorption intensities over the whole wavelength range, especially from 400 to 500 nm, indicating its better light absorption capacity induced by the improved film quality, which may make a great contribution to *J*_sc_ of PEOz-passivated PSC. In addition, the two films exhibit a similar typical absorption edge of ~785 nm that corresponds to the bandgap of MA_0.7_FA_0.3_PbI_3−x_Cl_x_ (1.58 eV) [45]. The results demonstrate that the introduction of PEOz does not affect the band gap of MA_0.7_FA_0.3_PbI_3−x_Cl_x_ perovskite, which is further verified by the XRD measurements. As shown in Figure 3b, both films exhibited diffraction peaks at 14.18°, 28.50°, and 31.98°, indexed to the (110), (220), and (310) planes of the MA_0.7_FA_0.3_PbI_3−x_Cl_x_ crystals, respectively [44,45]. In addition, the intensities of the dominant diffraction peaks of MA_0.7_FA_0.3_PbI_3−x_Cl_x_ film are clearly increased with the treatment of PEOz, and neither diffraction signals for PbI_2_ nor PEOz are detected, which indicates the better crystallinity of MA_0.7_FA_0.3_PbI_3−x_Cl_x_ perovskite with fewer grain boundaries and intergranular defects. From these results, we can conclude that the PEOz treatment can beneficially passivate the surface and boundary defects, and hence boost the film quality of MA_0.7_FA_0.3_PbI_3−x_Cl_x_ without affecting its crystal structure.

To further verify the above inference, we measured the steady-state photoluminescence (PL) spectra and time-resolved photoluminescence (TRPL) spectra for perovskite films with and without PEOz treatment. The perovskite films were grown directly on insulated glass substrates. As shown in Figure 3c, the PEOz-treated perovskite film exhibits stronger PL intensity around 790 nm compared to the pristine one, indicating the enhanced radiative recombination and weaker nonradiative recombination in it. This can be further evidenced by the TRPL results in Figure 3d, in which the semi-quantitative information about the nonradiative recombination of charge carriers is calculated with a bi-exponential decay function. Obviously, the PEOz-treated film yields the average carrier lifetime (*τ*_ave_) of 205.93 ns, which is higher than that of the pristine perovskite film (178.43 ns). The elongated PL decay time of the former means a lower defect density and hence superior photoelectric properties for the PEOz-treated perovskite films. This is primarily ascribed to the strong coordinate bonding between PEOz and the perovskite, which beneficially passivates the defects on the perovskite surfaces and boundaries by effective repression of nonradiative recombination paths.

### 3.3. Effect of PEOz on the PSCs Photovoltaic Performance

**Figure 4:**
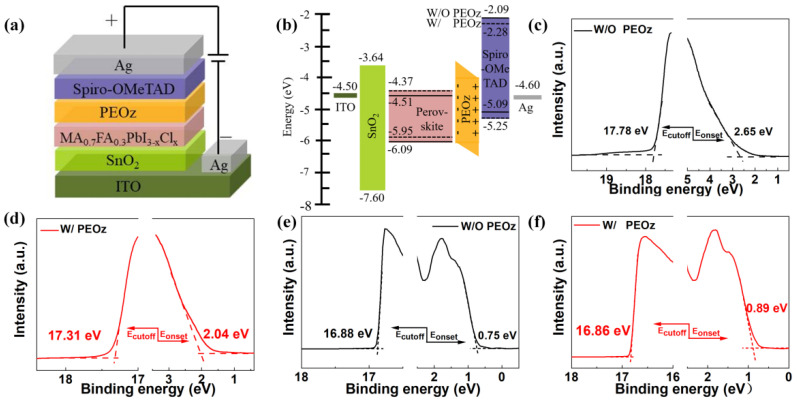
(**a**) Schematic device structure and (**b**) energy band diagrams of corresponding materials used in the PSCs (ITO/SnO_2_/perovskite/PEOz/spiro−OMeTAD/Ag). (**c**) UPS spectra of the perovskite film without and (**d**) with PEOz passivation, (**e**) Spiro−OMeTAD thin film without and (**f**) with PEOz passivation show the VBM onset (E_onset_) and photoemission cut−off energy boundary (E_cutoff_) values.

The device structure used in this experiment is shown in Figure 4a and the polymer PEOz is introduced between the perovskite light-absorbing layer and the hole transport layer. The dipole effect of PEOz makes the HOMO energy level difference between the perovskite layer and the HTL 1.0 eV is reduced to 0.7 eV (Figure 4b) [54,55]. The lower the energy level difference, the more beneficial it is to inhibit electron–hole recombination and accelerate hole extraction, which is of great significance to the collection of photogenerated carriers and the improvement of battery performance.

To further explore the influence of PEOz on the HOMO energy of the perovskite layer and the HTL, we conducted ultraviolet photoelectron spectroscopy (UPS) tests. As indicated in Figure 4c,d, the HOMO energy level of the pristine perovskite film and PEOz-treated perovskite film is 6.09 eV and 5.95 eV, respectively, according to the formula of HOMO = 21.22 − (Ecutoff − Eonset) [13]. The HOMO energy levels of the Spiro-OMeTAD film without and with a PEOz under layer are 5.09 eV and 5.25 eV, respectively (Figure 4e,f). The simultaneous change of HOMO energy levels of perovskite and Spiro-OMeTAD could promote the favorable band regulation between the perovskite and Spiro-OMeTAD layer with the corresponding energy barrier reduced by 0.3 eV, while a better hole transport with restrained interfacial non-radiative recombination can be expected, which further facilitates the increase in FF and Voc in the cell [13,56].

**Figure 5:**
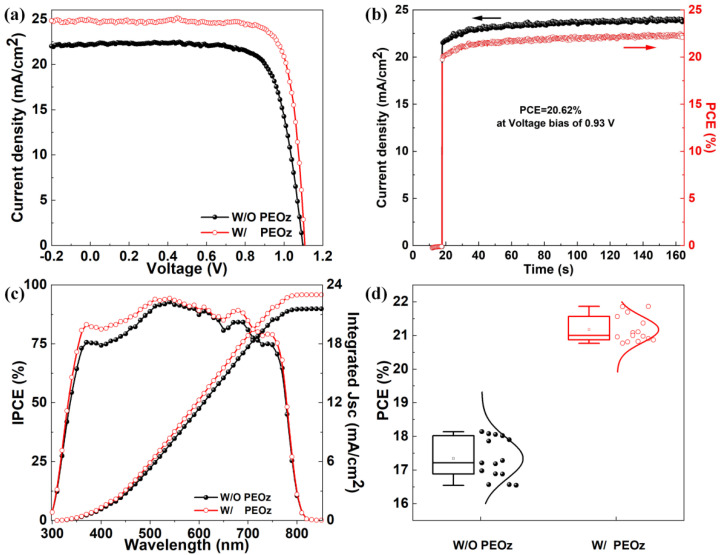
(**a**) Champion performance J-V characteristics of the control device and with PEOz−based device. (**b**) Steady output characteristics of the PEOz−based device. (**c**) IPCE curves and integrated current density and of (**d**) statistic result of PCE for the control devices and PEOz−based devices.

Taking into account the defect passivation effect of PEOz on the uncoordinated Pb2+ of the perovskite film, the perovskite/HTL HOMO energy level is more matched, which has a beneficial effect on the extraction and transport of holes; we use PEOz solution to process perovskite films, which has been used to prepare a device with a structure of ITO/SnO_2_/MA_0.7_FA_0.3_PbI_3−x_Cl_x_/PEOz/Spiro-OMeTAD/Ag. Figure 5a shows that the device obtained by modifying the perovskite with 1 mg/mL PEOz solution exhibits an optimally high efficiency of 21.86%, which is about 21% higher than the 18.14% of the comparative sample. Moreover, the detailed performance parameters are shown in Table 1, a Jsc of 24.88 mA/cm^2^, a Voc of 1.11V, an FF as high as 79.32% far above the control devices, a Jsc of 22.22 mA/cm^2^, a Voc of 1.09 V, and an FF of 74.56%. These results verify the above-mentioned dual effect of PEOz. In Figure 5b and Figure S2, the steady output current densities were examined for the PSC treated by PEOz and the pristine PSC under the condition of maximum power points (MPPs, 0.93 and 0.92 V) and are 22.17 and 19.27 mA/cm^2^, with the corresponding PCE of 20.62% and 17.72%, respectively. The two stable PCE values are close to those calculated from the J-V curve, which not only effectively confirms the reliability of the J-V test, but also convincingly demonstrates that the introduction of PEOz has significantly improved the performance of PSC. In addition, the incident photo-to-current conversion efficiency (IPCE) measurements were performed. The results in Figure 5c manifest that the PSC treated by PEOz exhibits higher photon-electron transformation efficiencies over the whole response wavelength region, revealing a better perovskite/HTL interface for the extraction and transmission of charge carriers. What is more noteworthy is that, especially from 350 nm to 450 nm, PSC treated with PEOz has more obvious photo-to-current conversion efficiency, which is almost the same as the UV-vis spectra of Figure 3a, which more effectively proves that PEOz can improve the film quality and enhance its light absorption capacity.

During the preparation of the device, it is affected by the gas atmosphere, temperature, and heating time of the cross-solvent solution in the glove box, resulting in the problem of poor repeatability of the device. To evaluate the reproducibility of PSCs, we measure the PCE of 15 PSCs from three batches fabricated with pristine and treated by PEOz PSCs. The statistical results in Figure 5d and Table 1 show that the average PCE value is increased from 17.34 ± 0.80 to 21.17 ± 0.69, after processing with PEOz. Meanwhile, the distribution deviation of PSCs treated by PEOz is reduced by 16% compared with the pristine PSCs. In addition, as shown in Figure S3 and Table 1, the average *J*_sc_, *V*_oc_ and FF of the PSCs treated by PEOz are about 24.70 ± 0.18 mA/cm^2^, 1.10 ± 0.01 V and 77.35 ± 1.97 higher than that of the pristine PSCs, which have an average *J*_sc_, *V*_oc_ and FF of about 20.77 ± 1.45 mA/cm^2^, 1.04 ± 0.05 V and 71.44 ± 3.12, respectively. These data are in accordance with the J-V characteristics shown in Figure 5a, indicating a good reproducibility and a better performance of PEOz-treated devices. The hysteresis behavior of the PSCs was also improved after surface passivation by PEOz (Figure S4). Moreover, the hysteresis index (HI) is usually used to measure the hysteresis effect in PSCs. The specific formula is shown in Formula (1) [57].

(1) $$\mathrm{HI}=\frac{J_{\mathrm{scan}}+(0.8V_{\mathrm{oc}})-J_{\mathrm{scan}}-(0.8V_{\mathrm{oc}})}{J_{\mathrm{scan}}+(0.8V_{\mathrm{oc}})}$$

where *J*_scan_ + (0.8*V*_oc_) and *J*_scan_ − (0.8*V*_oc_) represent the photocurrent density of 0.8 V in forward scanning and reverse scanning, respectively. The results show that the device with the PEOz layer achieved the lowest hysteresis index (2.6%), compared to the control device (4.9%). The improvement of hysteresis may be ascribed to the passivation layer of PEOz which decreased the surface traps of MA_0.7_FA_0.3_PbI_3−x_Cl_x_, thereby reducing the nonradiative recombination. This also proves that the PEOz at the grain boundary shown by the SEM in Figure 2 helps to reduce the boundary defects and related energy loss of the perovskite film.

**Figure 6:**
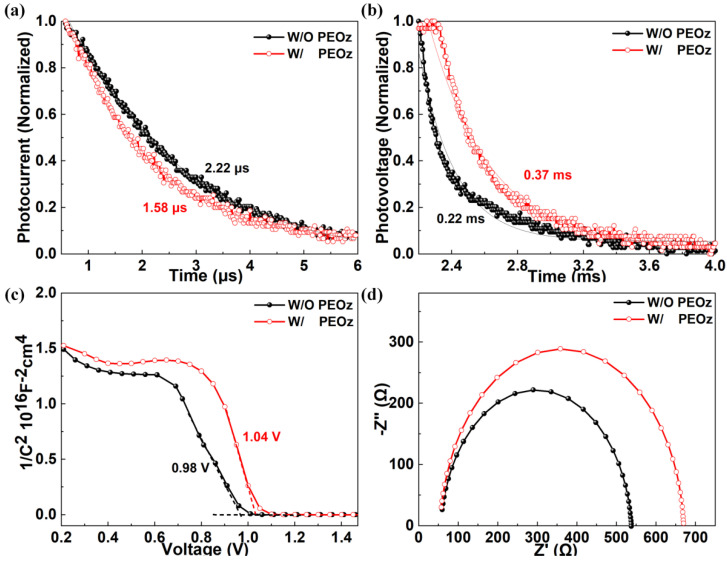
(**a**) TPC, (**b**) TPV and (**c**) M−S measurements of the control PSCs and PEOz−based PSCs. (**d**) The corresponding Nyquist plots measured at a forward bias of 1.2 V dark condition of the control PSCs and PEOz−based PSCs.

The photocurrent (TPC) and photovoltage (TPV) tests are used to analyze the carrier dynamics of PSCs with PEOz and W/O PEOz [15,44]. The TPC results tested under short-circuit conditions are shown in Figure 6a, the decay time of the PSCs introduced with PEOz is 1.58 μs, which is 0.64 μs lower than the 2.22 μs of the control devices. A smaller decay time indicates more efficient carrier extraction and transmission, which may be the reason for the obvious increase in *J*_sc_ in Figure 5a. Meanwhile, Figure 6b shows the TPV results measured in the open-circuit state, compared with the basic devices, the photovoltage decay value of the PEOz-treated devices is increased from 0.22 ms to 0.37 ms. This result supports the weaker recombination of charge carriers in it, indicating the better perovskite crystallization quality by the perovskite film modification of the PEOz layer.

We further tested the Mott–Schottky (M-S) and the electrochemical impedance spectra (EIS) of the PSCs with W/O PEOz in the dark state, so as to explore the carrier recombination behavior on a deeper level. In Figure 6c, C is the dark state capacitance, and V_bi_ is the built-in potential. Since the introduction of PEOz can make the energy levels of the perovskite/HTL more matched, the PSCs treated with PEOz have a higher V_bi_, which can provide a greater separation force for photogenerated carriers, thereby inhibiting their recombination. It can be seen from the figure that the V_bi_ of the PSCs treated by PEOz has increased from 0.98 V to 1.04 V. A higher V_bi_ is conducive to the improvement of the device *V*_oc_, which is also consistent with the *V*_oc_ result on the J-V curve of Figure 5a. The EIS in Figure 6d is mainly divided into two parts, the first part is that the large semicircle of the low-frequency part is related to the recombination resistance (Rrec) of the PSCs, and a large Rrec means a low carrier composite probability [15,45,58]. It can be seen that the Rrec of PSCs with PEOz is increased from 263 Ω to 538 Ω, indicating that PEOz can make the separation of photogenerated carriers of PSCs easier, resulting in the weaker internal recombination of PSCs. This is mainly because PEOz can effectively passivate defects in MA_0.7_FA_0.3_PbI_3−x_Cl_x_ films, which is also consistent with the TRPL analysis results in Figure 3d. The second part is the series resistance (Rs) of the high frequency part, which is also an important parameter. Compared with the basic device (307 Ω), the PEOz-treated device has lower Rs (50 Ω), which shows that PEOz can effectively reduce the series resistance of PSCs, to improve the FF value and improve the performance of the battery.

### 3.4. Effect of PEOz on the PSCs Stability

**Figure 7:**
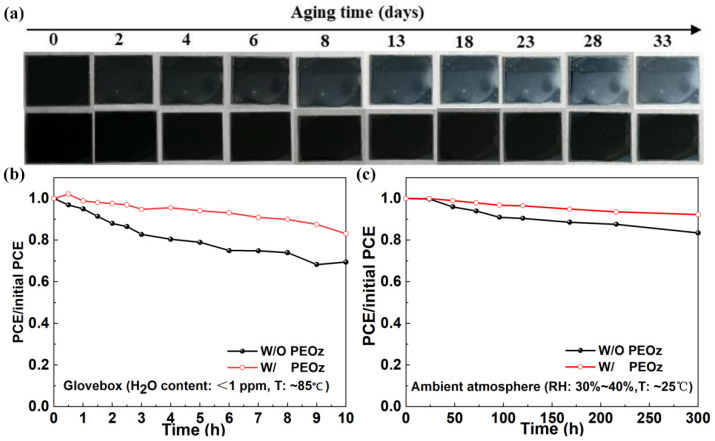
(**a**) Photographs of the MA_0.7_FA_0.3_PbI_3−x_Cl_x_ films with and without PEOz passivation layer during exposure to a relative humidity of 30~40% over 29 days. (**b**) Thermal stability and (**c**) Wet stability results of the device with and without PEOz, respectively.

Finally, in order to study the effect of PEOz modification on the stability of the perovskite device, We observed the optical image of the degradation of the perovskite film at 30~40% relative humidity (RH) within 33 days (Figure 7a). It could be observed that small white dots started to appear in the pristine perovskite film the next day, these dots continued to expand until the black film was completely hydrolyzed into white at about 33 days. By contrast, it was until 33 days that the white dots begun to exist on the PEOz passivated perovskite film, showing higher water stability. Meanwhile, as shown in Figure S6, the contact angles of water on the perovskite film W/O PEOz and with PEOz are measured to be 60.92°, and 39.62°, respectively, suggesting that PEOz is hygroscopic, which may absorb water molecules in ambient atmosphere preferentially before the perovskite does, slowing down the decomposition of the perovskite layer.

In addition, we compared the thermal stability of an unpackaged battery in a glovebox with water content of less than 1ppm and a temperature of 85 °C. As shown in Figure 7b, the device with PEOz can still maintain an initial efficiency of 83.13% after heating for 10 h, while the device W/O PEOz shows only 79.05% of its initial efficiency under the same storing condition. This may be due to poor crystal quality instability of MA_0.7_FA_0.3_PbI_3−x_Cl_x_ exposed at high temperatures [59]. We also placed the unpackaged battery in a drying cabinet with a humidity of 30~40% and a temperature of 25 °C. In Figure 7c, the device modified by PEOz can still maintain an initial efficiency of 92.29% after storage for 300 h. By contrast, under the same conditions, the initial efficiency of the device W/O PEOz dropped to 83.46%. These results indicate that PEOz can ensure the stability of PSCs in a humid and hot environment, and effectively increase the service life of the device. This is primarily due to the PEOz wrapped around the perovskite grains since the decomposition of PSCs generally starts from the grain boundaries. With the preservation of grain boundaries to not being exposed to ambient atmosphere, the decomposition of PSCs is effectively suppressed [60].

## 4. Conclusions

In summary, we demonstrate for the first time, the successful application of multifunctional dipolar polymer PEOz as a simultaneous interfacial modifier and passivation agent at the perovskite/HTL interface in n-i-p planar PSCs. It is found that the lone pair electrons of the oxygen atoms in PEOz can bond with the uncoordinated lead ions on the perovskite surfaces and GBs, which effectively passivates the defect sites, reduces the trap density in perovskite film, and thereby facilitates the absorption ability and photoluminescence lifetime of the resulting PSCs. We also illustrate that the dipole of PEOz provides an optimized interface band alignment and boosts the hole transport/extraction couple with low non-radiation recombination at the perovskite/HTL interface. Moreover, the hygroscopic property of PEOz on top of the perovskite film contributes to the high stability of the PSCs during operation and under ambient air atmosphere. Consequently, with the above three benefits, we realized an optimized PSC efficiency of 21.83%, with a Voc of 1.11 V, Jsc of 24.88 mA/cm^2^, and FF of 0.79, much higher than the control device. Remarkably, the non-encapsulated PEOz-based PSCs show outstanding long-term stability against moisture and thermal stresses, retaining 92.29% or 83.13% of its initial PCE value after 300 h of storage in ambient air with relative 30~40% humidity (RH) air, or being heated at 85 °C for 10 h in a glovebox, respectively. As a result, these findings contribute to providing an important guide for effectively improving the performance and environmental stability of the perovskite materials and the resulting optoelectronic devices.

## Figures and Tables

**Table 1:** Photovoltaic parameters of the control devices and PEOz-based devices.

		*J*_sc_ (mA/cm^2^)	*V*_oc_ (V)	FF (%)	PCE (%)
W/O PEOz	best	22.22	1.09	74.56	18.14
	average	20.77 ± 1.45	1.04 ± 0.05	71.44 ± 3.12	17.34 ± 0.80
W/PEOz	best	24.88	1.11	79.32	21.86
	average	24.70 ± 0.18	1.10 ± 0.01	77.35 ± 1.97	21.17 ± 0.69

## Data Availability

The data presented in this study are available on request from the corresponding author.

## Supplementary Materials

The following supporting information can be downloaded at: https://www.mdpi.com/article/10.3390/polym14132748/s1, Figure S1: J-V characteristics of best-performing control device and devices fabricated with different concentrations of PEOz under AM 1.5G illumination (100 mW cm^−2^); Figure S2: Steady-state photocurrent and PCE output characteristics of the control device; Figure S3: Statistical results of (a) Jsc, (b) Voc and (c) FF for the device without and with PEOz, respectively; Figure S4: J–V curves with different scanning directions of the PSCs (a) without and (b) with PEOz layer, respectively; Figure S5: Water contact angles of the MA_0.7_FA_0.3_PbI_3−x_Cl_x_ films (a) without and (b) with PEOz deposition, respectively; Table S1: Photovoltaic parameters of best-performing control device and device prepared with different concentrations of PEOz.
