# Supplementary material for: Interfacial Dipole poly(2-ethyl-2-oxazoline) Modification Triggers Simultaneous Band Alignment and Passivation for Air-Stable Perovskite Solar Cells
Source: Polymers (Basel). 2022 Jul 5;14(13):2748. doi: 10.3390/polym14132748 (PMC9269119; doi:10.3390/polym14132748)
Supplement: Supplementary file 1 [file polymers-14-02748-s001.zip › polymers-1792786-supplementary.pdf]

Supporting Information

Interfacial Dipole Poly(2-ethyl-2-oxazoline) Modification Triggers Simultaneous Band Alignment and Passivation for Air-Stable Perovskite Solar Cells

He Xi [1,*], Zhicheng Song [2], Yonggang Guo [3], Weijia Zhu [1], Lisong Ding [1], Weidong Zhu [2], Dazheng Chen [2] and Chunfu Zhang [2,*]

[1] School of Advanced Materials and Nanotechnology, Xidian University, Xi'an 710126, China; 18329743263@163.com (W.Z.); d1446124526@126.com (L.D.)
[2] State Key Discipline Laboratory of Wide Band Gap Semiconductor, School of Microelectronics, Xidian University, Xi'an 710071, China; songzhicheng@spic.com.cn (Z.S.); wdzhu@xidian.edu.cn (W.Z.); dzchen@xidian.edu.cn (D.C.)
[3] Qinghai Huanghe Hydropower Development Co., Ltd., Xi'an Solar Power Branch, Xi'an 710000, China; guoyonggang@spic.com.cn
* Correspondence: hxi@xidian.edu.cn (H.X.); cfzhang@xidian.edu.cn (C.Z.)

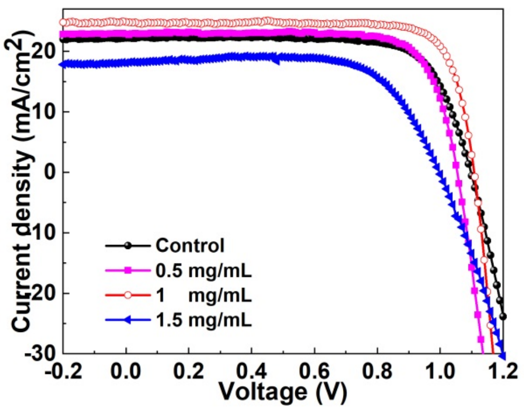

Figure S1. *J*–*V* characteristics of best-performing control device and devices fabricated with different concentrations of PEOz under AM 1.5G illumination (100 mW cm^{-2}).

Table S1. Photovoltaic parameters of best-performing control device and device prepared with different concentrations of PEOz.

	J_{sc} (mA/cm^2)	V_{oc} (V)	FF (%)	PCE (%)
Control	22.22	1.09	74.56	18.14
0.5 mg/mL	22.90	1.05	77.35	18.66
1 mg/mL	24.88	1.11	79.32	21.86
1.5 mg/mL	18.13	1.00	71.46	12.93

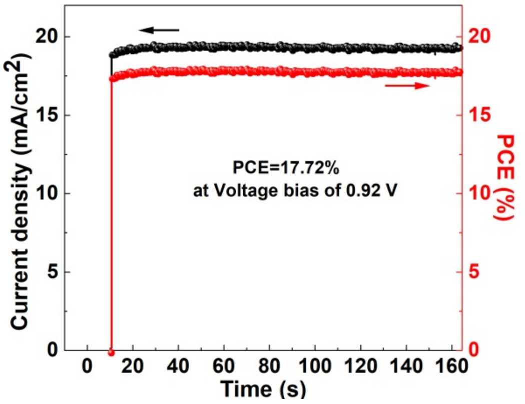

Figure S2. Steady-state photocurrent and PCE output characteristics of the control device.

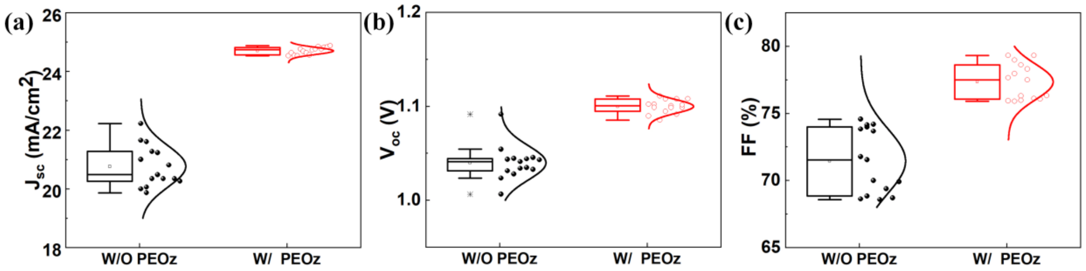

Figure S3. Statistical results of (a) J_{sc}, (b) V_{oc} and (c) FF for the device without and with PEOz, respectively.

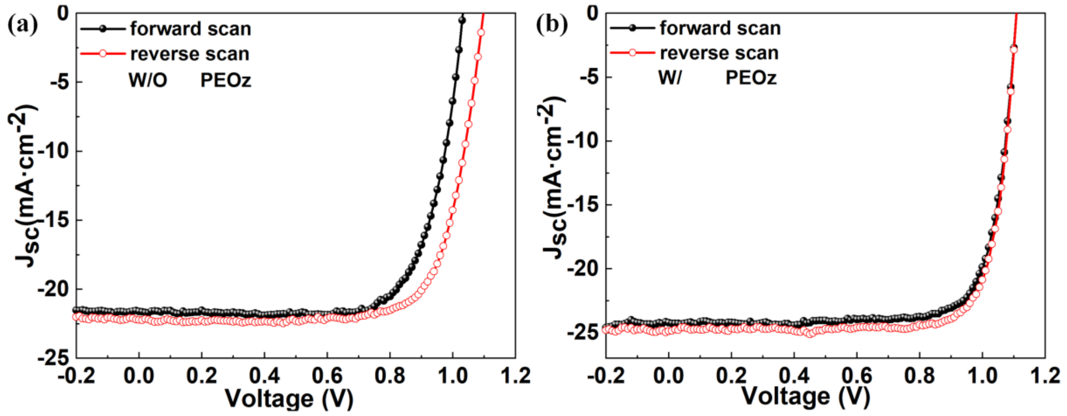

Figure S4. *J–V* curves with different scanning directions of the PSCs (a) without and (b) with PEOz layer, respectively.

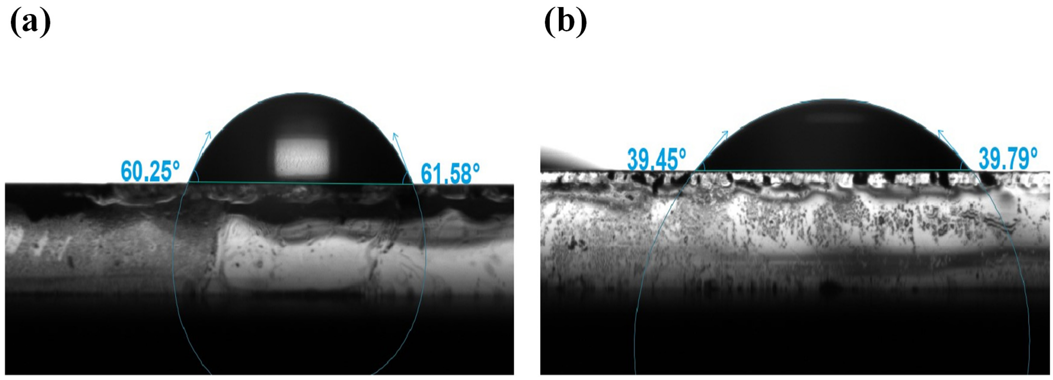

Figure S5. Water contact angles of the $MA_{0.7}FA_{0.3}PbI_{3-x}Cl_x$ films (a) without and (b) with PEOz deposition, respectively.
